# Modulation of Intrathymic Sphingosine-1-Phosphate Levels Promotes Escape of Immature Thymocytes to the Periphery with a Potential Proinflammatory Role in Chagas Disease

**DOI:** 10.1155/2015/709846

**Published:** 2015-08-04

**Authors:** Ana Flávia Nardy, Leonardo Santos, Célio Geraldo Freire-de-Lima, Alexandre Morrot

**Affiliations:** ^1^Instituto de Microbiologia, Universidade Federal do Rio de Janeiro, 21941-590 Rio de Janeiro, RJ, Brazil; ^2^Instituto de Biofísica Carlos Chagas Filho, Universidade Federal do Rio de Janeiro, 21941-590 Rio de Janeiro, RJ, Brazil

## Abstract

The sphingosine-1-phosphate (S1P) system regulates both thymic and lymph nodes T cell egress which is essential for producing and maintaining the recycling T cell repertoire. Infection with the protozoan parasite *Trypanosoma cruzi* induces a hormonal systemic deregulation that has impact in the thymic S1P homeostasis that ultimately promotes the premature exit of immature CD4^−^CD8^−^ T cells expressing TCR and proinflamatory cytokines to peripheral lymphoid organs, where they may interfere with adaptive immune responses. In what follows, we review recent findings revealing escape of these immature T cells exhibiting an activation profile to peripheral compartments of the immune system in both experimental murine and human models of Chagas disease.

## 1. Introduction

T lymphocytes are key players in acquired immunity and have a lineage commitment characterized by expression of the T cell receptor (TCR), which has a vital role in recognizing pathogen antigens during the development of host resistance to infections [1]. The activation and differentiation of T cells depend on the TCRs being specific for exogenous antigens but not mounting an autoimmune response against self-antigens and generating a collateral response. This quality control of the immune system is performed during the maturation of T cell precursors in the thymus [2, 3].

The thymus is the primary lymphoid organ in which T cell precursors derived from the bone marrow undergo cell differentiation process consisting of the sequential expression of multiple lymphocyte differentiation genes and rearrangement of the T cell receptor (TCR) genes. During thymic maturation, thymocytes express TCRs, some of which interact with peptides presented by molecules of the major histocompatibility complex (MHC) on the surface of the thymic stromal cells. These interactions determine the positive and negative selection events that are crucial components of the program of terminal thymocyte differentiation [4–6].

During intrathymic development, thymocytes begin to express on their membranes the TCR/CD3 complex together with CD4 and CD8 coreceptors, thus becoming double-positive (DP) thymocytes, distributed throughout most of the cortical region of the organ. At this phase of intrathymic maturation of thymocytes, the generation of a highly diverse TCR repertoire produces many T lymphocytes expressing TCRs that recognize “self-antigens.” These autoreactive T lymphocytes are negatively selected in the thymus as part of the process called central tolerance. In this process, the self-reactive lymphocytes die by apoptosis, while a small percentage of positively selected cells move to the medulla of the thymus where their differentiation proceeds [6–8].

During the course of their differentiation, thymocytes develop into T cells expressing high densities of TCR/CD3 and they become simple positive (SP) for one or another (but not both) of the coreceptors CD8 or CD4, which recognize, respectively, peptides complexed with class I and class II MHC molecules. These naïve T cells ultimately leave the thymus to form part of the repertoire of peripheral T cells [1, 9]. They are exported from the thymus under the control of the lipid mediator sphingosine-1-phosphate (S1P) [10–12]. Sphingosine-1-phosphate is a biologically active sphingolipid derivative critical to the signaling pathways involved in the traffic of leukocytes [10, 13, 14].

The tissue concentration of S1P increases in several inflammatory conditions such as asthma and autoimmune diseases and this lipid agonist engages and activates a family of G-protein coupled receptors (S1P1–S1P5) [15–17]. Several groups have demonstrated the importance of S1PRs in the trafficking of leukocytes mediating effector responses in the immune system. Their findings indicate a key role of the S1P-S1PRs axis in the development and maintenance of immunity [18, 19].

## 2. Fine-Tuned Metabolic Regulation of Sphingosine-1-Phosphate

Sphingolipids are essential lipids rich in cholesterol that are concentrated in microdomains known as “lipid rafts” or “lipid platforms” on the plasma membrane. These lipids can be rapidly metabolized upon activation of an enzymatic cascade that converts sphingolipids such as sphingomyelin and glycosphingolipid complexes to ceramide and subsequently to sphingosine, two sphingosine kinases (SphK1 and SPHK2) and then phosphorylate sphingosine to sphingosine-1-phosphate [17, 20, 21].

Sphingosine-1-phosphate has both cell-extrinsic and intrinsic activities affecting homeostasis and cellular function [22]. Much emphasis has been given to the extrinsic function of S1P in the immune system, which was recognized through studies of the immunosuppressive agent, FTY720, a drug mediator proved capable of binding to and blocking sphingosine-1-phosphate receptors (S1PRs) [23]. FTY720 induces lymphopenia by causing sequestration of lymphocytes in the lymph nodes, thus blocking the egress of mature thymocytes to the periphery [24, 25].

The tissue levels of S1P are determined not only by its rate of biosynthesis but also by its rate of degradation. It is constantly produced by most cells and is irreversibly degraded by S1P lyase or dephosphorylated by S1P phosphatases [26–28]. In most tissues including lymphoid organs, S1P levels are extremely low. Exceptions are the blood and lymph, in which S1P levels are generally high, ranging from submicro to micromolar concentrations, respectively [29, 30]. The S1P levels in serum arise mainly from its production by endothelial cells, while the high levels in plasma are contributed by the erythrocytes [31, 32].

Deletion of the genes encoding both of the kinases, SphK1 and SPHK2, results in embryonic lethality due to the absence of S1P. In addition, conditional deletion of these two genes results in deficiency in circulating S1P. However deletion of either one of the two genes is without effect, showing that these kinases have redundant functions [29, 33].

## 3. Expression of the Specific G Protein Coupled Receptors for S1P and the Regulation of Cellular Traffic in the Immune System

The discovery of the “orphan receptor”-associated G protein gene originally known as endothelial differentiation gene 1 (EDG1) opened a new frontier in our understanding of the mechanism of action of S1P [34]. Since then, S1P has been shown to be the ligand for five different members of this “orphan receptor” family, S1P1–S1P5 [35]. These receptors mediate several cellular functions through associated heterotrimeric G proteins (*α*I, *α*q, or *α*12/13) and are expressed by most cells of the immune system. However, there is heterogeneity in terms of their pattern of expression among immune cells [36].

Although S1PRs are present in other physiologic systems, S1P3–S1P5 are mainly limited to the immune system. T cells express S1P1 and S1P4 [12, 37–39], while mast cells and macrophages express S1P1 and S1P2 [40–45]. Expression of S1P1 is also found in B lymphocytes and dendritic cells [46–50]. The primary function of most S1PRs is to regulate the migratory responses of cells by inducing proteins with Rac GTPase activity [36, 51]. S1P signaling plays a role both in the migration or homing of immune cells to lymphoid organs and in their egress into the blood and lymph, a topic that has received much attention recently [52–54]. The S1P gradient between lymphoid tissues, which have low levels of S1P, and their vascular compartments, which have high levels of S1P, is a key factor determining the egress of leukocytes from lymph nodes and thymus into the blood [32]. The signaling pathways activated by S1P1 in response to this gradient of S1P control not only the egress of T and B lymphocytes from lymph nodes but also the exit of mature T lymphocytes and natural killer T cells (NKT) from the thymus to vascular compartments [10, 12, 14].

S1P-mediated chemotaxis via S1P1 is dependent on the concentration of SIP:* in vitro* studies have demonstrated that low concentrations promote S1P chemotaxis, while high concentrations tend to inhibit it [54, 55]. It appears that high S1P levels stimulate ubiquitin-dependent lysosomal membrane protein sorting and degradation, which causes breakdown of S1P1 [56]. Interestingly, elevated concentrations of the synthetic agonist of S1PRs, FTY720, are also highly effective in inducing internalization, ubiquitination, and degradation of S1P1 [23, 57–59].

## 4. Disruption of S1P Homeostasis Promotes Thymocyte Precursor Release and Organ Atrophy in Chagas Disease

In most vertebrates, acute short-term stress signals induced by infectious pathogens are responsible for evoking host innate defense responses [60]. If a pathogen persists chronically the stress signals can cause the host immune response to be suppressed, thus increasing susceptibility to the microorganism; this results at least in part from a shift from T helper 1-mediated cellular immunity to T helper 2-mediated humoral immunity. These stress signals are the result of release of neurotransmitters, hormones, and cytokines secreted during inflammatory responses [61].

In Chagas disease, infection by* T. cruzi* generates an inflammatory syndrome mediated by TNF-*α* in the acute phase. This cytokine activates the hypothalamus-pituitary-adrenal (HPA) axis leading to release of stress hormone corticosterone. The primary consequence is atrophy of the thymus leading to severe reduction in thymic cell numbers, followed by a reduction in the thymic output of T cells to the periphery [62, 63].

Although thymic atrophy occurs in infections caused by several pathogens, the impact of this trait on thymic central tolerance has only recently been clarified. We have demonstrated that the alterations in the thymic microenvironment induced by* T. cruzi* infection do not affect the key elements needed for intrathymic negative selection of maturing thymocytes during thymopoiesis [64]. Nevertheless, we have observed that in severe atrophy of the thymus the number and frequency of developing extrathymic thymocytes bearing low TCR levels increase markedly during the acute phase [65].

Moreover, we found that the immature thymocytes released into the periphery (subverting the process of negative selection) acquired similar activated phenotype to that described for activated effector cells and single-positive memory cells, suggesting a possible imbalance in the mechanism controlling thymocyte exit to the periphery [64]. As the signaling pathway mediated by sphingosine-1 phosphate (S1P) through its receptors is responsible for the egress of mature thymocytes, we investigated whether some modulation of the sphingosine-1-phosphate (S1P) pathway was responsible for the early release of thymocytes in thymic atrophy [66].

The gene expression profile of the enzymes involved in the S1P pathway in the* T. cruzi* infected thymus during acute phase in fact showed a reduction of sphingosine-1-phosphate accompanied by reduced expression of the activating kinases SPHK1/2 and upregulation of the inactivating phosphatase SGPL1. However, the S1P levels in the sera of infected and normal mice were similar [66]. These findings indicate that the gradient of S1P level from the thymus to the blood of* T. cruzi* infected mice becomes steeper upon infection and this may promote thymocyte egress. Since high concentrations of S1P are needed to stimulate ubiquitin-dependent lysosomal membrane protein sorting and degradation the breakdown of S1P1 this feedback mechanism should not be active in the infected thymus because of the reduced S1P concentration.

Interestingly, analysis of the expression of the S1P receptor in developing thymocytes indicated a substantial upregulation of S1P1 and S1P3 expression in immature CD4^−^CD8^−^ T cells upon* T. cruzi* infection, suggesting that some S1P-mediated pathway could also contribute to the premature exit of these cells [66]. The upregulation of S1P receptors should increase the sensitivity of thymocytes to S1P-mediated chemotaxis in the atrophic thymus. Using an* ex vivo* Transwell migration assay, we found that the sensitivity of the CD4^−^CD8^−^ cells to S1P-mediated chemotaxis increased during* T. cruzi* infection [66].

These findings together indicate that the export of developing CD4^−^CD8^−^ thymocytes could be favored by a disturbance of the physiological levels of S1P in the thymus during* T. cruzi* infection. In fact, we found that when we blocked the S1P receptors with FTY720 in infected mice we inhibited the escape of these cells to the periphery thus restoring physiological levels of CD4^−^CD8^−^ thymocytes [66].

Furthermore, when we assessed the differentiation status of the CD4^−^CD8^−^ thymocytes released during* T. cruzi* infection, we found that these immature cells (present in peripheral lymph nodes) exhibited a significant increase in the expression of the cytokines IL-17 and TNF*α* upon polyclonal stimulation [66]. The findings we have described correlate with the presence in patients with the indeterminate or cardiac clinical forms of Chagas disease of increased numbers of circulating CD4^−^CD8^−^ T cells exhibiting an activated phenotype as defined by the expression of activation marker, HLA-DR [64].

## 5. Concluding Remarks

Overall our studies indicate that infection with* Trypanosoma cruzi* promotes thymic alterations, due in part to the effects of modulation of the S1P-S1P1 receptor axes on intrathymic CD4^−^CD8^−^ T cells. As a result, thymocytes undergoing differentiation become prematurely chemotactically responsive to S1P [66]. These double-negative thymocytes are therefore able to emigrate from the thymus before undergoing the negative selection process necessary for self-tolerance. Moreover these immature T cells that escaped to periphery have an activated phenotype in both experimental murine and human models of Chagas disease [64].

The presence of these cells bearing TCRs in the periphery may have potential implications for disease outcome. In other systems, these cells have been shown to have pathogenic properties: they are able to recognize antigens and signal through their TCR in an MHC-independent manner [67, 68]. Importantly, as demonstrated in patients with systemic lupus erythematous and myasthenia gravis, there is a direct correlation between CD4^−^CD8^−^ T cells and the development of human autoimmune diseases, suggesting a role for these cells in autoimmune responses [69].

It is also possible that the early egress of undifferentiated CD4^−^CD8^−^ T cells plays a role in the immunopathologic events in Chagas disease by altering adaptive immune responses, since they produce proinflammatory cytokines when activated. In addition to these various phenomena, our results indicate a direct link between the changes in the level of the CD4^−^CD8^−^ T cell subset and the severity of myocardial lesions in human Chagas disease, thus identifying a potential clinical marker of disease progression [66].
